# METTL3 Promotes the Proliferation and Mobility of Gastric Cancer Cells

**DOI:** 10.1515/med-2019-0005

**Published:** 2019-03-02

**Authors:** Sen Lin, Jianing Liu, Wen Jiang, Peng Wang, Chao Sun, Xuexiang Wang, Yuan Chen, Hongbo Wang

**Affiliations:** 1 Department of Gastroenterology, The second hospital of Shandong University, 250033 Jinan, Shandong, China; 2 Thyroid/Pancreatic surgery, The second hospital of Shandong university, 250033 Jinan, Shandong, China; 3 Central Research Laboratory, The second hospital of Shandong University, 250033 Jinan, Shandong, China

**Keywords:** Proliferation, Migration, Invasion, Apoptosis, AKT

## Abstract

Methyltransferase-like 3 (METTL3) was originally known to be responsible for N6-methyladenosine (m6A) modification of mRNA. Recent studies have found that METTL3 plays important roles in a variety of tumors by regulating the translation of oncogenes. However, the functional and regulating mechanisms of METTL3 in human gastric cancer have not yet been understood. Here we knocked down METTL3 in human gastric cancer cell lines, AGS and MKN45, by using shRNA transfection. RT-qPCR assay and western blotting verified the effectiveness of RNA interference on mRNA and protein levels, respectively. Then we found that METTL3 knockdown inhibited cell proliferation, migration and invasion in AGS and MKN45 cells. Moreover, METTL3 knockdown decreased Bcl2 and increased Bax and active Caspase-3 in gastric cancer cells, which suggested the apoptotic pathway was activated. Mechanistic investigation suggested that METTL3 led to inactivation of the AKT signaling pathway in human gastric cancer cells, including decreased phosphorylation levels of AKT and expression of down-stream effectors p70S6K and Cyclin D1. In conclusion, our study reveals that down-regulation of METTL3 inhibits the proliferation and mobility of human gastric cancer cells and leads to inactivation of the AKT signaling pathway, suggesting that METTL3 may be a potential target for the treatment of human gastric cancer.

## 1 Introduction

Gastric cancer is the fifth most common malignancy in the world and approximately 951,600 new cases were diagnosed in 2012 [1]. Despite the decline in the death rate due to gastric cancer in recent years, it is still the third leading cause of cancer deaths [2, 3]. At present, most diagnosed gastric cancer patients are already in the advanced stages, accompanied by malignant hyperplasia, extensive infiltration, lymph node metastasis or distant metastasis [2, 3]. The treatment of gastric cancer is still based on surgical resection and chemotherapy, which leads to many problems such as side effects, recurrence and metastasis [4]. Although significant advances have been made in the study of the molecular mechanisms of gastric cancer, such as oncogene and tumor suppressor mutations [5], individual abnormal genome changes [6], and identification of cancer stem cells [7], there are still great limitations in clinical treatment, and the mortality rate of gastric cancer is still high. Therefore, the elucidation of new mechanisms related to the pathogenesis of gastric cancer is crucial for the development of effective targeted therapies for human gastric cancer.

N6-methyladenosine (m6A) is the most common modification of mRNAs and lncRNAs [8]. m6A modification mainly occurs on adenine in the RRACH sequence (R = G or A; H = A, C or U) [9, 10, 11]. Increasing evidences suggest that m6A modification is involved in the regulation of RNA stability, localization, splicing and translation [12, 13]. It has been found that m6A may affect numerous biological processes, including development, immunity, DNA damage response, tumor formation or metastasis, stem cell renewal, biological rhythm, cell differentiation, cell cycle, and so on [14, 15, 16]. m6A modification is achieved by the methyltransferase complexes, including Methyltransferase-like 3 (METTL3), methyltransferase-like 14 (METTL14) and Wilms’ tumor 1associating protein (WTAP) [17, 18]. Recent studies have shown that METTL3 promotes the translation of a variety of oncogenes involved in the proliferation, survival and invasion of human cancers [19, 21]. However, the function and mechanism of METTL3 in gastric cancer have not been studied yet.

In this study, we investigated the function of METTL3 in human gastric cancer by using RNA interference technology. We explored the action mechanism of METTL3 by focusing on the AKT/mTOR signaling pathway. Our results suggest that METTL3 may be a potential therapeutic target for the treatment of human gastric cancer.

## 2 Materials and methods

### 2.1 Cell culture

Human gastric cancer AGS and MKN45 cells were purchased from the Cell Bank of The Chinese Academy of Sciences (Shanghai, China). The cells were cultured in Dulbecco’s Modified Eagle’s Medium-High glucose (DMEM) (Sigma-Aldrich, USA) supplemented with 10% fetal bovine serum (FBS) (Gibco) and incubated at 37 °C in 5% CO2.

### 2.2 shRNA transfection

shRNA targeting METTL3 (shRNA) was designed and constructed by Genewiz (Beijing, China)using the pcDNA3.1 vector for stable transfection. When growing in logarithmic phase, gastric cancer cells were transfected with shMETTL3 interference plasmid by using Lipofectamine 6000 liposome according to the manufacturer’s protocol. A scrambled RNA was used as a negative control (NC) in all experiments. The sequences of shRNAs were as follows:

shMETTL3: 5’-GCTGCACTTCAGACGAATT-3’

shNC: 5’-AATTCTCCGAACGTGTCACGT-3’.

### 2.3 RT-qPCR

Total RNA of gastric cancer cells transfected with shNC or shMETTL3 was extracted by using the Ultrapure RNA extraction kit. cDNA was synthesized by using the Prime-Script RT Master Mix kit (Takara, Tokyo, Japan) following the instructions. mRNA expression of METTL3 was determined by fluorescence quantitative PCR. 2^−ΔΔCt^ method was used to quantify the mRNA expression. The PCR primers were as follows:

METTL3-F: 5’-CAAGCTGCACTTCAGACGAA-3’,

METTL3-R: 5’-GCTTGGCGTGTGGTCTTT-3’;

β-actin-F: 5’-ACTGGAACGGTGAAGGTGAC-3’,

β-actin-R: 5’-AGAGAAGTGGGGTGGCTTTT -3’.

### 2.4 Proliferation assays

CCK-8 assay: After transfection with shNC or shMETTL3 for 24 h, gastric cancer AGS and MKN45 cells were seeded into a 96-well plate at 2000 cells/well. Cell viability was determined by using a CCK-8 kit (Dojindo, Japan). Each group had three repeat wells and the experiment was repeated three times.

Colony formation: After transfection with shNC or shMETTL3 for 24 h, gastric cancer AGS and MKN45 cells (approximately 500 of each) were seeded into 10-cm culture dishes. The cells were then continually cultured for 14 days. After staining with 0.1% crystal violet for 30 min, cells were then imaged and counted under a microscope.

### 2.5 Scratch assay

Gastric cancer cells were transfected with shNC or shMETTL3 for 24 h and then seeded into six-well plates with DMEM medium (10% FBS). After cells grew to 50% confluence, a scratch was formed in the cell monolayer by using a 200 μl sterile pipette tip. After removing detached cells, the remaining cells were cultured as normal for 48 h. Images of scratches were taken at 0 h, 24 h, and 48 h. The percentage of wound closure, defined as (wound closure/initial wound area)×100%, was calculated.

### 2.6 Cell invasion analysis

Cell invasion was assessed by using transwell Matrigel invasion chambers (BD science, USA). The pore size of transwell membrane was 8 μm. Gastric cancer cells transfected with shNC or shMETTL3 were counted and added in the transwell insert at 2.5×10^4^ in 500 μl of serum-free medium. The lower chamber was added along with 500 μl of medium containing 10% FBS. After 24 h of incubation at 37 °C, the residual cells on the upper surface were removed by cotton swab. Invasive cells on the lower surface were fixed with methanol and dyed by 0.1% crystal violet. The invasive cells were counted in 5 random fields and imaged with a microscope.

### 2.7 Western blotting

The expression of target proteins was assessed by western blot. After transfection for 24 h, the cells were starved for 24 hours inmedium with 1% FBS. Then total proteins from gastric cancer cells were extracted by using RIPA buffer with 1% protease inhibitor. Proteins were concentration-measured by BCA method and subjected to SDS-PAGE at 20 μg total protein per lane. Immunodetection was performed with standard techniques. Antibodies to Bcl2, Bax, Active-Caspase-3, AKT, p-AKT, p70S6K, Cyclin D1 and α-Tubulin were purchased from Abcam (Shanghai, China). The visualization of protein bands was performed by using the ECL development system. ImageJ software was used to analyze the protein densities.

### 2.8 Statistical analysis

All experiments were performed in triplicate. Comparison between two groups was performed using unpaired two-tailed Student’s t test or one-way analysis. Prism 7 (Graph-Pad Software) was used for all statistical analysis. *P*<0.05 was considered significant.

## 3 Results

### 3.1 Down-regulation of METTL3 inhibits the proliferation of gastric cancer cells

**Figure 1:**
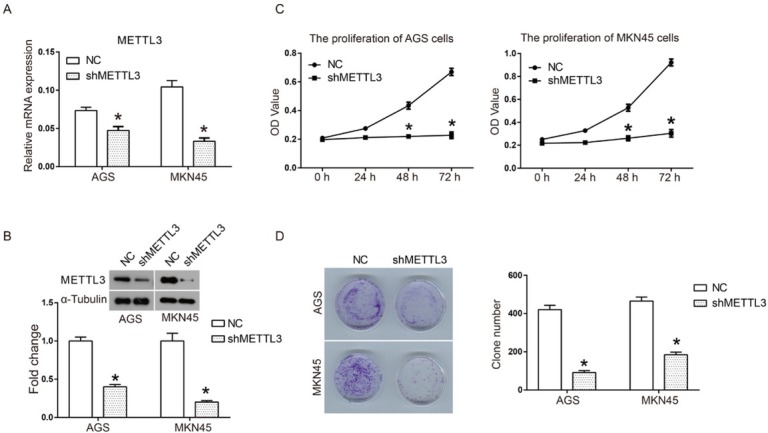
Down-regulation of METTL3 inhibited cell proliferation of human gastric cancer cells. shMETTL3 interference plasmid was constructed and introduced into human gastric cancer cells. A scrambled shRNA was used as a negative control (NC) in all experiments. (A) The interference efficiency of METTL3 was detected by using RT-qPCR; (B) Cell proliferation of AGS and MKN45 was detected by CCK8 assay; (C) and (D) Colony formation of AGS and MKN45 cells after transfection with shMETTL3 or shNC. All experiments were performed in triplicate. *P<0.05.

In order to study the effect of METTL3 on human gastric cancer *in vitro*, a METTL3-targeted shRNA was designed and constructed. As shown in Fig 1A qRT-PCR assay results suggested that the mRNA expression of METTL3 was significantly down-regulated after shMETTL3 was introduced into the human gastric cancer cell lines AGS and MKN 45. Moreover, western blot results also confirmed the effectiveness of RNA interference (Fig 1B) Sequentially, we analyzed the impact of METTL3 knockdown on the proliferation of gastric cancer cells. As shown in Fig 1B compared to the fast growth in the NC group, the proliferation of AGS and MKN 45 cells was almost completely blocked after transfection with shMETTL3, and cell viability exhibited a significant difference at 48 h and 72 h. In addition, the influence of METTL3 knockdown on colony formation of human gastric cancer was also investigated. As shown in Fig 1C compared to the large number of cell colonies of the NC group, gastric cancer cells transfected with shMETTL3 only formed rare colonies. Quantified results of clone formation indicated that clone number was decreased from 420±23 of the NC group to 91±10 of the shMETTL3 group in AGS cells and 465±21 to 184±14 in MKN45 cells. Taken together, these data suggest that down-regulation of METTL3 by shRNA transfection significantly inhibits cell proliferation and colony formation by human gastric cancer *in vitro*.

### 3.2 Down-regulation of METTL3 inhibits cell mobility of human gastric cancer cells

**Figure 2:**
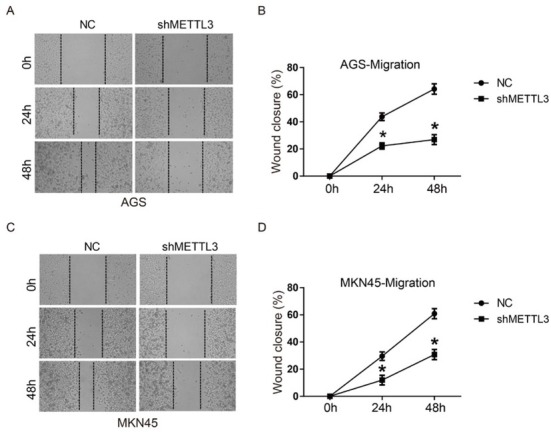
Down-regulation of METTL3 inhibited cell migration of human gastric cancer cells. AGS and MKN45 cells were transfected with shNC or shMETTL3 for 24 h and then subjected to a scratch assay for 48 h. (A) The images of scratch wounds in AGS cells at 0 h, 24 h and 48 h; (B) The curve of wound closure (%) in AGS; (C) The images of scratch wounds in MKN45 cells at 0 h, 24 h and 48 h; (B) The curve of wound closure (%) in MKN45. Wound closure (%) was defined as (wound closure area/initial wound area) ×100%. All experiments were performed in triplicate. *P<0.05.

**Figure 3:**
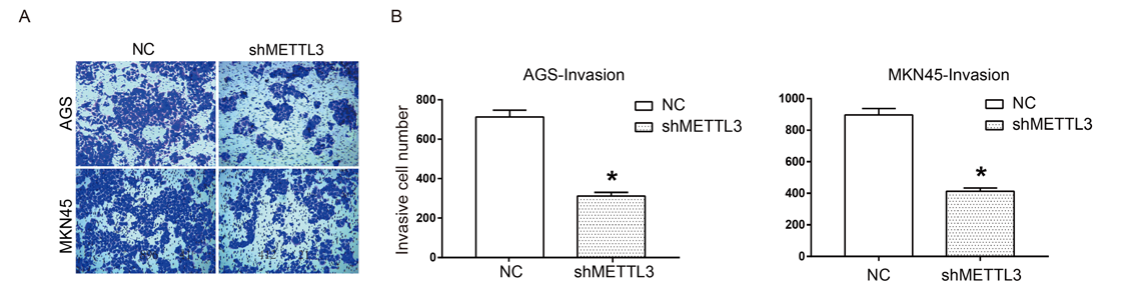
Down-regulation of METTL3 inhibited cell invasion of human gastric cancer. Cell invasion of AGS and MKN45 cells transfected with shNC or shMETTL3 was evaluated by using a transwell assay. (A) The images of invasive AGS and MNK45 cells; (B) Quantitative results of cell invasion in AGS and MKN45 cells. All experiments were performed in triplicate. *P<0.05.

In order to test whether METTL3 was involved in migration and invasion of human gastric cancer cells, a scratch assay and a transwell assay were performed. As shown in Fig 2, wound closure of shMETTL3 transfected cells was significantly inhibited compared to NC group in both AGS and MKN45 cells at 24 h and 48 h, suggesting that down-regulation of METTL3 could inhibit cell migration of gastric cancer. Transwell assay results in Fig 3 also indicated that invasive cell numbers of the shMETTL3 group were significantly less than that of the NC group in both AGS and MKN 45 cells. These data suggest that down-regulation of METTL3 significantly inhibits cell migration and invasion of human gastric cancer *in vitro*.

### 3.3 Down-regulation of METTL3 regulates apoptosis-related protein expression

**Figure 4:**
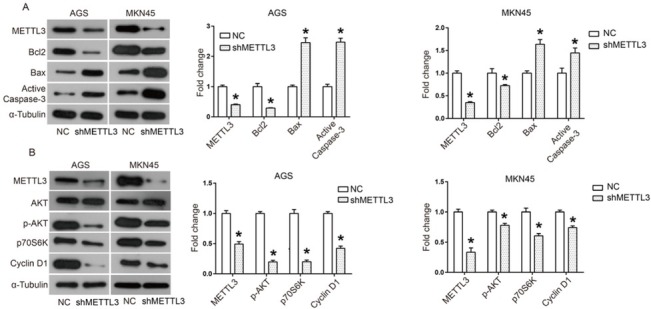
Down-regulation of METTL3 promoted apoptotic pathway and inactivated AKT signaling pathway (A) The expression of apoptosis-related proteins including Bcl2, Bax and Active Caspase-3 was analyzed by western blot and ImageJ software; (B) AKT signaling pathway including AKT, p-AKT, p70S6K and Cyclin D1 was analyzed by western blot and ImageJ software. All experiments were performed in triplicate. *P<0.05.

Bcl2/Bax and caspase cascade pathways play distinct roles in intrinsic apoptotic pathways. When apoptosis is triggered, Bax moves to the mitochondrial outer membrane to promote the opening of the permeability transition (PT) pore [22]. Sequentially, cytochrome C will be released to the cytoplasm and further activates the caspase cascade to induce apoptosis [22]. Bcl2 plays an anti-apoptosis role by blocking the opening of the PT pore through competitively combining with Bax [22]. Here through western blotting we demonstrated that down-regulation of METTL3 increased the expression of Bax and active-casapase-3, while it decreased the expression of Bcl2 (Fig 4A) These data suggest that down-regulation of METTL3 activates this apoptosis-related pathway.

### 3.4 Down-regulation of METTL3 inactivates the AKT signaling pathway in human gastric cancer cells

In order to determine the action mechanism of METTL3 in human gastric cancer, we investigated the effect of METTL3 knockdown on the AKT signaling pathway. When the AKT pathway is activated, the phosphorylation level of AKT is elevated, which will further regulates numerous down-stream effectors, including up-regulating p70S6K and Cyclin D1 [23]. Here through western blotting we found that down-regulation of METTL3 reduced the phosphorylation level of AKT and the expression of p70S6K and Cyclin D1, suggesting that the AKT signaling pathway was inactivated (Fig 4B)

## 4 Discussion

For the first time, our study has revealed an oncogenic role for METTL3 in human gastric cancer *in vitro*. Through using RNA interference, we found that METTL3 knockdown significantly inhibited cell proliferation, migration and invasion in the human gastric cancer cell lines AGS and MKN45. In addition, METTL3 knockdown also decreased the expression of negative regulator of apoptosis Bcl2 and increased the positive regulators Bax and active caspase-3. Increasing evidence has shown that METTL3 plays important roles in a variety of tumors. For example, Mengnuo Chen et al. report that METTL3 is up-regulated and predicts a poor prognosis in human hepatocellular carcinoma (HCC) [21]. Knockdown of METTL3 reduces HCC cell proliferation and migration *in vitro* and inhibit HCC tumorigenicity and lung metastasis *in vivo* [21]. Minjun Du et al. also report that MiR-33a induces growth inhibition of NSCLC cells by targeting METTL3 mRNA [24]. However, in another study, METTL3 is identified as a tumor suppressor gene in renal cell carcinoma, inhibiting tumor proliferation, migration and the cell cycle [25]. These data suggest that METTL3 may play opposite roles in differentiated tumors, which may be due to the complexity of the tumor microenvironment and a high degree of tumor heterogeneity.

Previous studies have found that the action mechanisms of METTL3 in tumors can be divided into two modes: m6A-dependent and m6A-independent. For example, knockdown of METTL3 in HCC can reduce m6A methylation-mediated degradation of SOC2 and then enhance its expression, thus inhibiting the progression of liver cancer [21]. Xiaoli Cai et al. find that METTL3 enhances the expression of HBXIP through m6A modification, which further promotes the progression of breast cancer [26]. However, METTL3 is also found to up-regulate the expression of oncogenes through binding to gene promoters, or promoting the assembly of mRNA translation devices [19, 20].

In this study, we investigated the underlying mechanism of METTL3 in human gastric cancer by focusing on the AKT signaling pathway. Previous studies found that the AKT signaling pathway is a key pathway in the regulation of numerous important biological processes, such as promoting cell proliferation, survival and so on [23]. Our results indicate that down-regulation of METTL3 leads to inactivation of the AKT signaling pathway, including decreased p-AKT, p70S6K and Cyclin D1.

However, we failed to identify the direct target of METTL3 in human gastric cancer cell lines due to the experimental conditions. In another study, METTL3 was found to inhibit the development of renal cell carcinoma by inactivating the AKT signaling pathway, which is in opposition to our study [25]. These data suggest that the action mechanism of METTL3 in tumors still needs further study in the future.

In conclusion, our results suggest that down-regulation of METTL3 inhibits cell migration, migration and invasion in human gastric cancer in vitro, which may be mediated by modifying gene expression to promote apoptosis and inactivating the AKT signaling pathway. These data reveal that METTL3 may be a promising target for treatment of human gastric cancer.
